# Association between elevated C-reactive protein-triglyceride-glucose index and in-hospital major adverse cardiovascular events in acute coronary syndrome patients after percutaneous coronary intervention: a single-center prospective observational study

**DOI:** 10.3389/fendo.2025.1745588

**Published:** 2026-01-13

**Authors:** Hai Fan, Dan Xia, Jun Li, Xuebin Dong

**Affiliations:** 1 Department of Cardiology, Ma’anshan People’s Hospital Ma’anshan People’s Hospital Affiliated to Wannan Medical College, Maanshan, Anhui, China; 2 Department of Endocrinology, Ma’anshan People’s Hospital, Ma’anshan People’s Hospital Affiliated to Wannan Medical College, Maanshan, Anhui, China; 3 Department of Cardiology, Chongming Hospital Affiliated to Shanghai University of Medicine and Health Sciences, Shanghai, China

**Keywords:** acute coronary syndrome, C-reactive protein-triglyceride-glucose index, CTI, inflammation, major adverse cardiovascular events, metabolic disorders, percutaneous coronary intervention

## Abstract

**Objective:**

Inflammation and metabolic disorders play important roles in the pathogenesis of acute coronary syndrome(ACS). The C-reactive protein-triglyceride-glucose index(CTI) is a novel combined inflammatory-metabolic indicator. This study aimed to evaluate the association between CTI and in-hospital major adverse cardiovascular events(MACE) in ACS patients after percutaneous coronary intervention(PCI).

**Methods:**

This prospective observational study consecutively enrolled 300 patients who underwent PCI for ACS at our hospital from January 2023 to October 2025. C-reactive protein(CRP),triglyceride(TG), and fasting plasma glucose (FPG) were measured upon admission, and CTI values were calculated. Patients were divided into Q1-Q4 groups according to CTI quartiles. The primary endpoint was in-hospital MACE, defined as a composite of cardiac death, acute stent thrombosis, recurrent myocardial infarction, acute heart failure, and cardiogenic shock. Multivariate logistic regression analysis was used to assess the association between CTI and in-hospital MACE.

**Results:**

Among the 300 patients, 73 (24. 3%) experienced in-hospital MACE. Compared with the Q1 group, patients in the Q4 group had significantly higher incidence of MACE (10. 7% *vs* 44. 0%, P<0. 001). Multivariate analysis showed that, after adjusting for traditional risk factors, the highest CTI quartile group(Q4) remained significantly associated with MACE risk (adjusted OR 3. 28, 95%CI 1. 42-7. 56, P , 0. 005). For each standard deviation increase in CTI, the risk of MACE increased by 46% (OR 1. 46, 95%CI 1. 21-1. 76, P<0. 001). CTI demonstrated better predictive value for MACE (AUC, 0. 703, 95%CI 0. 641-0. 766) compared to CRP (AUC, 0. 610), TG (AUC, 0. 655), or FPG (AUC, 0. 678) alone (all P<0. 05). Subgroup analysis showed that CTI had stronger predictive ability in patients with ST-segment elevation myocardial infarction, diabetes, and multivessel disease.

**Conclusion:**

Elevated CTI levels after PCI in ACS patients are significantly associated with increased risk of in-hospital MACE. CTI may be an effective tool for evaluating short-term prognosis in ACS patients after PCI, providing reference for early risk stratification and enhanced monitoring.

## 1 Introduction

Acute coronary syndrome (ACS) is a group of clinical syndromes characterized by coronary atherosclerotic plaque rupture or erosion leading to thrombus formation, including unstable angina (UA), non-ST-segment elevation myocardial infarction (NSTEMI), and ST-segment elevation myocardial infarction (STEMI) (1). ACS is one of the leading causes of mortality and disability worldwide. Statistics show that approximately 7 million people die from coronary heart disease globally each year, while the number of coronary heart disease patients in China exceeds 11 million (2, 3). Percutaneous coronary intervention (PCI), as the main reperfusion strategy for ACS, has significantly improved clinical outcomes (4). However, despite continuous advances in modern PCI techniques and widespread use of antiplatelet and statin therapies, approximately 10-15% of ACS patients still experience major adverse cardiovascular events (MACE) after PCI (5). Therefore, early identification of high-risk patients and implementation of risk stratification to guide individualized treatment are of significant clinical importance for further improving outcomes in ACS patients.

Inflammatory responses play a crucial role in the formation, progression, and rupture of coronary atherosclerotic plaques (6). C-reactive protein (CRP) is an acute-phase protein synthesized by the liver and serves as a sensitive marker reflecting systemic inflammatory status (7). Multiple studies have confirmed that elevated CRP levels are closely associated with adverse outcomes in ACS patients (8). Research by Liuzzo et al. demonstrated that serum CRP levels in unstable angina patients were significantly higher than those in stable angina patients, and elevated CRP levels were an independent predictor of cardiovascular events in unstable angina patients (9). The Fragmin during Instability in Coronary Artery Disease (FRISC) trial study showed that elevated CRP levels at admission in ACS patients could predict increased long-term mortality (10).

In addition to inflammatory factors, metabolic disorders are also important factors influencing the occurrence and prognosis of ACS. Triglycerides (TG) are lipid indicators closely associated with cardiovascular disease. Elevated TG levels not only promote atherosclerosis progression but also increase the risk of thrombosis by affecting platelet function, coagulation systems, and endothelial function (11). The Pravastatin or Atorvastatin Evaluation and Infection Therapy-Thrombolysis In Myocardial Infarction(PROVE IT-TIMI) 22 study showed that elevated TG levels in ACS patients were independent predictors of cardiovascular event recurrence, even when LDL-C was controlled to target levels (12). Abnormal fasting plasma glucose (FPG), especially stress hyperglycemia, has been confirmed to be significantly associated with increased in-hospital mortality and complication rates in ACS patients (13). Kosiborod et al. found that elevated blood glucose levels at admission in acute myocardial infarction patients were associated with increased in-hospital mortality, regardless of whether they had a history of diabetes (14). Hyperglycemia can exacerbate myocardial ischemia-reperfusion injury through multiple mechanisms, including increased oxidative stress, endothelial dysfunction, enhanced inflammatory response, and thrombotic tendency (15).

In recent years, composite indices integrating multiple metabolic and inflammatory markers have shown superior predictive value compared to single indicators in cardiovascular disease risk assessment (16). For example, the triglyceride-glucose (TyG) index has been proven effective in predicting coronary heart disease severity and prognosis (17). As a surrogate marker of insulin resistance, the TyG index not only correlates with the degree of coronary artery calcification but also predicts the risk of MACE in ACS patients after PCI (18). Similarly, inflammation-related indicators such as the neutrophil-to-lymphocyte ratio and platelet-to-lymphocyte ratio (PLR) have also been confirmed to effectively predict outcomes in ACS patients (19). Additionally, a prospective study demonstrated that the albumin-CRP ratio, which integrates albumin and CRP, has superior predictive ability for in-hospital mortality in acute myocardial infarction patients compared to CRP or albumin alone (20).

Based on the limitations of individual indicators and the advantages of composite indices, we propose a new composite indicator C-reactive protein-triglyceride-glucose index (CTI), which integrates information from three aspects, CRP reflecting inflammatory status, TG reflecting lipid metabolism, and FPG reflecting glucose metabolism. This multidimensional composite index theoretically may provide more comprehensive risk assessment than single indicators (21). In the pathogenesis of coronary heart disease, inflammatory responses, lipid deposition, and glucose metabolic disorders interact and collectively promote the occurrence and development of atherosclerosis (22). CRP can directly participate in atherosclerosis development through mechanisms such as activating the complement system, promoting monocyte adhesion and chemotaxis, and inducing endothelial cell expression of adhesion molecules (23). Hypertriglyceridemia can increase the penetration of LDL particles into the endothelium, promote macrophage foam cell formation, and enhance inflammatory responses (24). Hyperglycemia can damage vascular endothelium and promote inflammatory responses through pathways including the generation of advanced glycation end products (AGEs), increased oxidative stress, and protein kinase C activation (25). The synergistic effect of these three factors may play an important role in ACS occurrence and post-PCI prognosis.

Clinically, although various risk scoring systems exist for evaluating ACS patient prognosis, such as the Global Registry of Acute Coronary Events (GRACE) score and Thrombolysis In Myocardial Infarction(TIMI) score (26, 27),these scoring systems are primarily based on clinical characteristics and basic biochemical indicators and do not fully integrate biomarker information reflecting multi-system metabolic disorders. In comparison, CTI, as a simple, readily available, and cost-effective composite indicator, may provide new insights for risk stratification in ACS patients after PCI (28). Furthermore, this composite indicator is theoretically less affected by random variations and detection errors compared to single indicators, potentially offering higher stability and reliability (29).

To date, what is well-established in this research area is that inflammatory markers (particularly CRP), lipid parameters (including TG), and glucose metabolism indicators (such as FPG) individually contribute to cardiovascular risk prediction in ACS patients. Additionally, the value of certain composite indices like the TyG index and inflammation-based ratios has been demonstrated. However, several important gaps remain in the current knowledge (1), Despite the known pathophysiological interconnections between inflammation, lipid metabolism, and glucose metabolism in ACS, no composite index has yet integrated markers from all three of these pathways (2); The potential synergistic effect of combining CRP, TG, and FPG into a single index for predicting short-term outcomes in ACS patients after PCI has not been investigated (3); Whether such a combined index would provide superior risk stratification compared to established individual biomarkers or existing risk scores remains unknown; and (4) The optimal approach to integrate these three distinct biomarkers into a clinically useful composite index has not been determined. These knowledge gaps highlight the need for developing and validating novel multidimensional risk assessment tools that capture the complex interplay between inflammation and metabolic factors in ACS.

This study employs a prospective observational study design to investigate the association between CTI and in-hospital MACE in ACS patients after PCI, and to evaluate the value of this index as a prognostic prediction tool. Through this research, we hope to provide a new tool for risk assessment in ACS patients after PCI and scientific evidence for the development of subsequent intervention measures.

## 2 Methods

### 2.1 Study design and population

This prospective observational study was conducted in the Department of Cardiology at our hospital, consecutively enrolling patients who underwent PCI treatment for ACS from January 1, 2023, to October 31, 2025. The inclusion criteria were as follows (1), age ≥18 years (2); diagnosis of ACS based on the latest European Society of Cardiology guidelines (30), categorized as follows:For STEMI, a) typical acute chest pain or equivalent symptoms (such as dyspnea, epigastric pain, or arm pain) lasting ≥20 minutes; b) ST-segment elevation ≥1 mm in two or more contiguous leads or new left bundle branch block; c) elevated cardiac biomarkers (cardiac troponin I above the 99th percentile upper reference limit). For NSTEMI, a) typical acute chest pain or equivalent symptoms lasting ≥20 minutes; b) absence of ST-segment elevation; c) elevated cardiac biomarkers (cardiac troponin I above the 99th percentile upper reference limit). For Unstable Angina, a) typical acute chest pain or equivalent symptoms lasting ≥20 minutes; b) ECG changes (ST-segment depression ≥0. 5 mm, or T-wave inversions ≥1 mm in two or more contiguous leads) without ST-segment elevation; c) no elevation of cardiac biomarkers (3). coronary angiography confirming significant coronary artery stenosis (≥70% diameter stenosis in at least one major epicardial coronary artery) (4); successful PCI procedure within 24 hours of hospital admission for STEMI patients or within 72 hours for NSTEMI/UA patients; and (5) provision of written informed consent.

**Figure 1:**
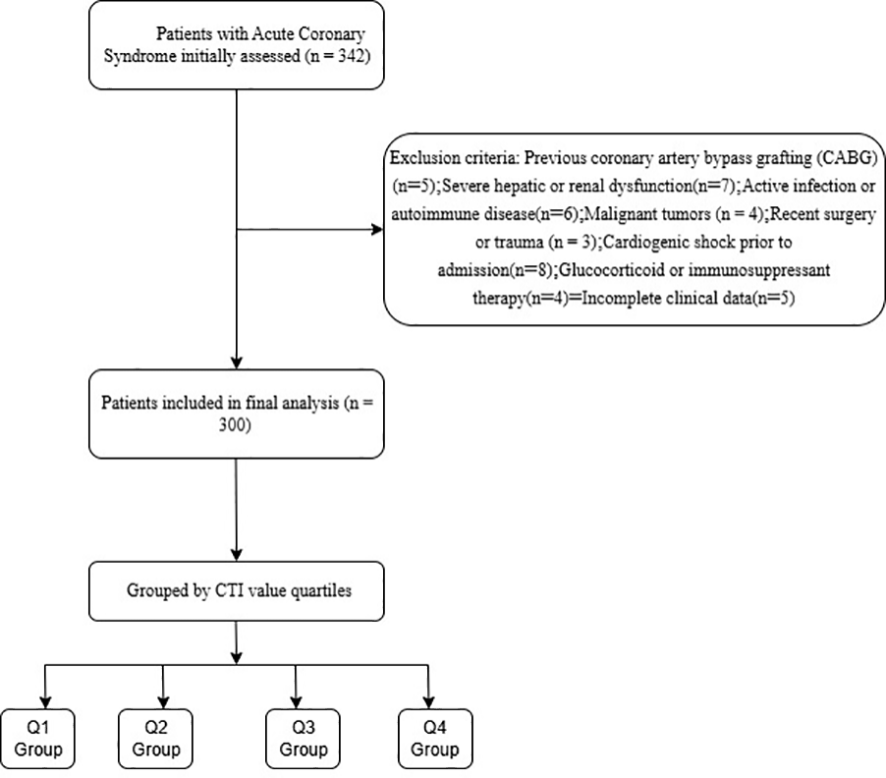
Study flow chart.

Exclusion criteria included (1), previous coronary artery bypass grafting (CABG) (2); severe hepatic or renal dysfunction (liver enzymes >3 times the upper limit of normal or estimated glomerular filtration rate <30 mL/min/1. 73m²) (3); active infection or autoimmune disease (4); malignant tumors (5); acute trauma or recent (<1 month) surgical history (6); glucocorticoid or immunosuppressant therapy (7); cardiogenic shock status prior to admission (8); incomplete data. The detailed flow diagram is shown in **Figure 1**.

Sample size calculation was performed based on the primary endpoint of in-hospital MACE. According to a previous study, the incidence of in-hospital MACE in ACS patients after PCI is approximately 10-15% (31). We hypothesized that patients in the highest CTI quartile would have at least a 2. 5-fold higher risk of MACE compared to those in the lowest quartile, with an estimated MACE rate of 30% in the highest CTI group. Using a two-sided alpha level of 0. 05 and a power of 80%, we calculated that a minimum of 264 patients (66 patients per quartile) would be required to detect this difference. Considering a potential dropout rate of approximately 10% due to incomplete data or loss to follow-up during hospitalization, we planned to enroll 300 patients in this study. The sample size calculation was performed using PASS software (version 15. 0, NCSS, LLC, Kaysville, Utah, USA). This study was approved by the Ethics Committee of Ma’anshan People’s Hospital (No:2025110521),and all patients signed informed consent forms.

### 2.2 Data collection

Demographic data, clinical characteristics, and laboratory test results were collected from all patients. Venous blood samples were drawn immediately upon admission to measure cardiac markers, and fasting blood samples were collected the following morning to determine complete blood count, liver and kidney function, FPG, lipid profile [total cholesterol(TC), low-density lipoprotein cholesterol (LDL-C), high-density lipoprotein cholesterol(HDL-C), and TG], and CRP. All biochemical parameters were measured using an automated biochemical analyzer.

Coronary angiography and PCI-related data were recorded, including the number of diseased vessels, lesion location, lesion characteristics (such as bifurcation, calcification, chronic total occlusion, etc.), number of stents, and stent length and diameter. Multivessel disease was defined as significant stenosis (stenosis degree ≥70%) in two or more major coronary arteries (left anterior descending, left circumflex, or right coronary artery). Complex lesions were defined as type B2/C lesions according to the American College of Cardiology/American Heart Association (ACC/AHA) classification criteria (32).

### 2.3 Calculation of CTI

In this study, CTI was used to assess participants’ levels of inflammation and insulin resistance. The formula for calculating CTI was 0. 412*Ln(CRP)+TyG (33), where TyG , Ln(fasting TG(mg/dL)*FPG(mg/dL))/2 (34). Higher CTI values indicate potentially more severe inflammation and insulin resistance in participants (35). Patients were divided into four quartile groups based on CTI values:Q1(<0. 62), Q2(≥0. 62,<0. 96), Q3(≥0. 96,<1. 34), and Q4(≥1. 34).

### 2.4 Definition of endpoints

The primary endpoint was in-hospital MACE, defined as a composite of the following events, cardiac death, acute stent thrombosis, recurrent myocardial infarction, acute heart failure, and cardiogenic shock. Cardiac death was defined as death resulting from an acute myocardial infarction, sudden cardiac death, death due to heart failure, death due to stroke of cardiac origin, death due to cardiovascular procedures, death due to cardiovascular hemorrhage, or any other death attributable to cardiovascular causes. This categorization was based on detailed review of hospital records, including laboratory tests, ECG changes, imaging findings, and documentation of the terminal event. When available, autopsy results were also considered in determining the cause of death. Acute stent thrombosis was defined as definite or probable stent thrombosis according to the Academic Research Consortium (ARC) criteria (36), occurring within 24 hours after the index procedure. Recurrent myocardial infarction was defined as recurrent chest pain lasting >30 minutes, with new electrocardiographic changes and a second rise of cardiac troponin values >20% from the previous nadir. Acute heart failure was defined as Killip class ≥II requiring intravenous diuretic therapy, with clinical symptoms and signs of pulmonary congestion confirmed by chest radiography or echocardiography. Cardiogenic shock was defined as systolic blood pressure <90mmHg lasting for more than 30 minutes, or requiring inotropic drugs and/or mechanical support to maintain blood pressure, along with evidence of end-organ hypoperfusion (cool extremities, oliguria <30 mL/hour, or altered mental status). All endpoint events were independently adjudicated by two experienced cardiologists, with disagreements resolved by a third expert. For mortality cases, the classification was performed according to the International Classification of Diseases, 10th Revision (ICD-10) coding system.

### 2.5 Statistical analysis

Continuous variables were presented as mean ± standard deviation or median (interquartile range) according to their distribution, and compared between groups using one-way analysis of variance (ANOVA) or Kruskal-Wallis test. Categorical variables were expressed as frequency (percentage) and compared using chi-square test or Fisher’s exact test.

Regarding data completeness, our analysis included only patients with complete data for all key variables, as patients with incomplete data were excluded from the study according to our predefined exclusion criteria. During the initial screening of 342 patients, 42 patients (12. 3%) were excluded due to incomplete data in one or more key variables. The final analysis dataset of 300 patients had no missing values; therefore, no imputation methods were needed. This complete case analysis approach was chosen to ensure the highest data quality and reliability of our findings.

Multivariate logistic regression analysis was used to evaluate the association between CTI and in-hospital MACE, calculating OR and 95%CI. The selection of variables for our progressive adjustment models followed established principles in cardiovascular epidemiology. Model 1 was intentionally unadjusted to show the crude association between CTI and in-hospital MACE. Model 2 adjusted for age, gender, and BMI, which represent fundamental non-modifiable demographic factors (age and gender) and a basic anthropometric measure (BMI). These factors are universal confounders in cardiovascular research as they significantly influence both inflammatory biomarkers and cardiovascular outcomes, independent of disease pathways. This minimal adjustment model allows assessment of the CTI-MACE relationship while controlling for basic patient characteristics that cannot be modified by clinical interventions. Model 3 was designed as a comprehensive adjustment model incorporating established cardiovascular risk factors (hypertension, diabetes, smoking), clinical history (previous myocardial infarction), disease presentation (ACS type), cardiac function (LVEF), coronary anatomy and procedural complexity (multivessel disease, complex lesions, number of stents), and metabolic and renal parameters (LDL-C, HDL-C, eGFR). These variables were selected based on (1), their established associations with cardiovascular outcomes in ACS patients as documented in previous studies (2), their potential to confound the relationship between inflammation-related indices and clinical outcomes, and (3) their availability in routine clinical practice, making our model clinically relevant and applicable. This hierarchical modeling approach allows for the evaluation of how the association between CTI and outcomes changes with progressive adjustment, helping to identify potential confounding pathways and assess the robustness of the primary relationship across different levels of adjustment. To explore the potential non-linear relationship between CTI values and the risk of in-hospital MACE, we employed restricted cubic spline (RCS) analysis with 4 knots placed at the 5th, 35th, 65th, and 95th percentiles of the CTI distribution. This approach allows for flexible modeling of potentially complex, non-linear associations while avoiding overfitting. The resulting spline function was incorporated into logistic regression models to visualize the shape of the relationship between CTI and MACE probability. We tested the non-linearity by comparing the model with the RCS terms to a model with only a linear term for CTI using a likelihood ratio test.

Receiver operating characteristic (ROC) curves were used to assess the ability of CTI and its individual components (CRP, TG, and FPG) to predict in-hospital MACE, calculating the area under the curve (AUC) for comparison. The optimal cut-off values for each predictor were determined using Youden’s index (sensitivity + specificity - 1). For each optimal cut-off value, we calculated the sensitivity, specificity, positive predictive value (PPV), negative predictive value (NPV), and accuracy for predicting in-hospital MACE. Sensitivity was calculated as the proportion of patients with MACE who were correctly identified by the test (true positives/[true positives + false negatives]). Specificity was calculated as the proportion of patients without MACE who were correctly identified (true negatives/[true negatives + false positives]). PPV was calculated as the probability that a patient with a positive test result actually had MACE (true positives/[true positives + false positives]). NPV was calculated as the probability that a patient with a negative test result actually did not have MACE (true negatives/[true negatives + false negatives]). Accuracy was calculated as the overall proportion of correct classifications ([true positives + true negatives]/total number of patients). To evaluate whether CTI offers advantages over established risk prediction tools, we compared the predictive performance of CTI against the GRACE score using DeLong’s test for comparing AUCs. Additionally, we calculated the net reclassification improvement (NRI) and integrated discrimination improvement (IDI) to evaluate the incremental predictive value of CTI when added to the GRACE score.

Pre-specified subgroup analyses were performed, including age (<65 years *vs* ≥65 years), gender, BMI (<25kg/m² *vs* ≥25kg/m²), diabetes status, ACS type (STEMI *vs* NSTEMI/UA), and multivessel disease. All statistical analyses were performed using SPSS 26. 0 and R 4. 3. 2 software, with P<0. 05 considered statistically significant.

## 3 Results

### 3.1 Baseline characteristics

This study included a total of 300 ACS patients, of whom 201 (67. 0%) were male, with a mean age of 63. 5 ± 11. 8 years. Baseline characteristics according to CTI quartiles are detailed in **Table 1**. Patients in higher CTI groups were older, had a higher proportion of females, and higher rates of elevated BMI, diabetes, and hypertension. Regarding laboratory parameters, patients in higher CTI groups exhibited higher levels of fasting plasma glucose, triglycerides, CRP, cardiac troponin I, and NT-proBNP, as well as lower HDL-C levels. Coronary angiography revealed that patients in higher CTI groups had higher proportions of multivessel disease and complex lesions, and received more implanted stents.

**Table 1:** Baseline characteristics of the study population grouped by CTI quartiles.

Characteristics	Total(n, 300)	Q1(n, 75)	Q2(n, 75)	Q3(n, 75)	Q4%(n, 75)	P-value
Demographic characteristics						
Age,years^,a^	63. 5 ± 11. 8	59. 3 ± 10. 7	62. 1 ± 11. 4	64. 8 ± 11. 5	67. 8 ± 12. 1	<0. 001
Female,n(%)^,c^	99(33. 0)	18(24. 0)	22(29. 3)	26(34. 7)	33(44. 0)	0. 006
BMI,kg/m²^,a^	25. 1 ± 3. 5	23. 6 ± 3. 2	24. 7 ± 3. 4	25. 8 ± 3. 5	26. 3 ± 3. 6	<0. 001
Medical history,n(%)						
Hypertension^,c^	176(58. 7)	35(46. 7)	41(54. 7)	47(62. 7)	53(70. 7)	<0. 001
Diabetes^,c^	97(32. 3)	15(20. 0)	20(26. 7)	27(36. 0)	35(46. 7)	<0. 001
Dyslipidemia^,c^	172(57. 3)	38(50. 7)	41(54. 7)	44(58. 7)	49(65. 3)	0. 042
Smoking^,c^	128(42. 5)	34(45. 3)	33(44. 0)	31(41. 3)	30(40. 0)	0. 618
Previous MI^,c^	32(10. 6)	6(8. 0)	7(9. 3)	9(12. 0)	10(13. 3)	0. 513
Previous PCI^,c^	41(13. 7)	9(12. 0)	9(12. 0)	10(13. 3)	13(17. 3)	0. 698
Clinical presentation,n(%)						
ACS Types						0. 004
STEMI^,c^	103(34. 3)	20(26. 7)	23(30. 7)	28(37. 3)	32(42. 7)	
NSTEMI^,c^	81(27. 0)	20(26. 7)	20(26. 7)	21(28. 0)	20(26. 7)	
UA^,c^	116(38. 7)	35(46. 7)	31(41. 3)	27(36. 0)	23(30. 7)	
Killip Class≥2^,c^	60(20. 0)	10(13. 3)	13(17. 3)	17(22. 7)	20(26. 7)	0. 015
LVEF,%^,a^	54. 8 ± 10. 2	57. 6 ± 9. 4	55. 9 ± 9. 8	53. 7 ± 10. 3	52. 0 ± 10. 6	<0. 001
Laboratory indicators						
White blood cell count,×10^9^/L^,a^	8. 7 ± 2. 9	7. 8 ± 2. 5	8. 3 ± 2. 7	8. 9 ± 2. 9	9. 6 ± 3. 1	<0. 001
Hemoglobin,g/L^,a^	137. 5 ± 17. 6	139. 8 ± 16. 8	138. 5 ± 17. 2	136. 9 ± 17. 7	135. 0 ± 18. 3	0. 085
Platelet count,×10^9^/L^,a^	229. 4 ± 62. 3	226. 1 ± 58. 9	228. 0 ± 61. 5	230. 2 ± 63. 4	233. 3 ± 65. 2	0. 726
Creatinine,μmol/L^,a^	79. 6 ± 28. 3	75. 8 ± 25. 4	77. 9 ± 26. 7	80. 5 ± 29. 1	84. 2 ± 31. 3	0. 038
eGFR,mL/min/1. 73m²^,a^	81. 9 ± 22. 4	86. 3 ± 20. 7	83. 5 ± 21. 5	80. 6 ± 22. 8	77. 2 ± 23. 9	0. 003
Total cholesterol,mmol/L^,a^	4. 65 ± 1. 15	4. 48 ± 1. 07	4. 59 ± 1. 11	4. 69 ± 1. 16	4. 82 ± 1. 21	0. 041
LDL-C,mmol/L^,a^	2. 91 ± 1. 02	2. 78 ± 0. 95	2. 87 ± 0. 98	2. 94 ± 1. 03	3. 05 ± 1. 09	0. 085
HDL-C,mmol/L^,a^	1. 03 ± 0. 30	1. 10 ± 0. 31	1. 06 ± 0. 30	1. 01 ± 0. 29	0. 96 ± 0. 28	<0. 001
Triglyceride,mmol/L^,a^	1. 82 ± 0. 97	1. 34 ± 0. 61	1. 65 ± 0. 79	1. 93 ± 0. 92	2. 34 ± 1. 19	<0. 001
Fasting plasma glucose,mmol/L^,a^	6. 59 ± 2. 51	5. 25 ± 1. 12	6. 01 ± 1. 68	6. 83 ± 2. 27	8. 27 ± 3. 35	<0. 001
HbA1c,%^,a^	6. 42 ± 1. 37	5. 79 ± 0. 82	6. 15 ± 1. 08	6. 58 ± 1. 29	7. 18 ± 1. 67	<0. 001
CRP,mg/L^,b^	3. 52 (1. 63-7. 25)	1. 12 (0. 68-1. 75)	2. 35 (1. 45-3. 56)	3. 97 (2. 63-5. 85)	9. 23 (6. 35-14. 17)	<0. 001
Troponin I,ng/mL^,b^	2. 05 (0. 33-8. 96)	1. 51 (0. 25-7. 21)	1. 76 (0. 29-8. 05)	2. 17 (0. 36-9. 53)	2. 58 (0. 42-11. 32)	0. 003
NT-proBNP,pg/mL^,b^	463 (205-1358)	378 (162-1046)	429 (187-1205)	485 (217-1437)	562 (246-1686)	<0. 001
CTI^,b^	0. 96 (0. 62-1. 34)	0. 47 (0. 35-0. 55)	0. 80 (0. 72-0. 88)	1. 14 (1. 05-1. 23)	1. 61 (1. 47-1. 89)	<0. 001
Angiographic characteristics,n(%)						
Number of diseased vessels						0. 002
Single vessel disease^,c^	143(47. 7)	43(57. 3)	38(50. 7)	34(45. 3)	28(37. 3)	
Double vessel disease^,c^	92(30. 7)	21(28. 0)	22(29. 3)	24(32. 0)	25(33. 3)	
Triple vessel disease^,c^	65(21. 7)	11(14. 7)	15(20. 0)	17(22. 7)	22(29. 3)	
Lesion location^,c^						
Left main coronary artery^,c^	15(5. 0)	3(4. 0)	3(4. 0)	4(5. 3)	5(6. 7)	0. 788
Left anterior descending artery^,c^	202(67. 3)	49(65. 3)	50(66. 7)	51(68. 0)	52(69. 3)	0. 842
Circumflex artery^,c^	95(31. 7)	21(28. 0)	23(30. 7)	24(32. 0)	27(36. 0)	0. 553
Right coronary artery^,c^	127(42. 3)	29(38. 7)	31(41. 3)	33(44. 0)	34(45. 3)	0. 547
Complex lesions (Type B2/C)^,c^	180(60. 0)	39(52. 0)	43(57. 3)	47(62. 7)	51(68. 0)	0. 013
Chronic total occlusion^,c^	31(10. 3)	6(8. 0)	7(9. 3)	8(10. 7)	8(10. 7)	0. 649
Bifurcation lesions^,c^	57(19. 0)	13(17. 3)	14(18. 7)	15(20. 0)	15(20. 0)	0. 707
Calcified lesions^,c^	67(22. 3)	13(17. 3)	15(20. 0)	18(24. 0)	21(28. 0)	0. 164
PCI characteristics						
Number of stents^,a^	1. 6 ± 0. 9	1. 4 ± 0. 8	1. 5 ± 0. 9	1. 7 ± 1. 0	1. 8 ± 1. 0	0. 002
Total stent length,mm^,a^	38. 7 ± 24. 8	35. 1 ± 22. 6	37. 2 ± 23. 9	40. 2 ± 25. 4	42. 3 ± 26. 7	0. 039
Stent diameter,mm^,a^	3. 02 ± 0. 45	3. 01 ± 0. 44	3. 02 ± 0. 45	3. 03 ± 0. 46	3. 01 ± 0. 45	0. 975
Use of IVUS/OCT^,c^	48(16. 0)	11(14. 7)	11(14. 7)	13(17. 3)	13(17. 3)	0. 899

BMI, Body Mass Index; MI, Myocardial Infarction; PCI, Percutaneous Coronary Intervention; ACS, Acute Coronary Syndrome; STEMI, ST-segment Elevation Myocardial Infarction; NSTEMI, Non-ST-segment Elevation Myocardial Infarction; UA, Unstable Angina; LVEF, Left Ventricular Ejection Fraction; eGFR, estimated Glomerular Filtration Rate; LDL-C, Low-Density Lipoprotein Cholesterol; HDL-C, High-Density Lipoprotein Cholesterol; HbA1c, Glycated Hemoglobin; CRP, C-Reactive Protein; NT-proBNP, N-terminal pro-B-type Natriuretic Peptide; CTI, C-Reactive Protein-Triglyceride-Glucose Index; IVUS, Intravascular Ultrasound; OCT, Optical Coherence Tomography. ^a^Normally distributed data are expressed as mean ± standard deviation; Groups were compared using one-way analysis of variance (ANOVA). ^b^Non-normally distributed data are expressed as median (interquartile range); Groups were compared using Kruskal-Wallis test. ^c^Categorical variables were expressed as frequency (percentage) and compared using chi-square test or Fisher’s exact test.

### 3.2 Relationship between CTI and MACE during hospitalization

Among the 300 patients, 73 (24. 3%) experienced in-hospital MACE, including 18 cases (6. 0%) of cardiac death, 15 cases (5. 0%) of acute stent thrombosis, 21 cases (7. 0%) of recurrent myocardial infarction, 29 cases (9. 7%) of acute heart failure, and 14 cases (4. 7%) of cardiogenic shock.

The occurrence of MACE across different CTI quartile groups is detailed in **Table 2**. From Q1 to Q4 groups, the incidence of MACE gradually increased (10. 7%, 16. 0%, 26. 7%, and 44. 0%, P<0. 001). The rates of individual endpoint events also increased with higher CTI quartiles, particularly cardiac death (P , 0. 007), recurrent myocardial infarction (P , 0. 012), and acute heart failure (P , 0. 002).

**Table 2:** Occurrence of MACE during hospitalization among different CTI quartile groups.

Events	Total(n, 300)	Q1(n, 75)	Q2(n, 75)	Q3(n, 75)	Q4(n, 75)	P-value
MACE^,c^	73(24. 3)	8(10. 7)	12(16. 0)	20(26. 7)	33(44. 0)	<0. 001
Cardiac death^,c^	18(6. 0)	1(1. 3)	3(4. 0)	5(6. 7)	9(12. 0)	0. 007
Acute stent thrombosis^,c^	15(5. 0)	2(2. 7)	3(4. 0)	4(5. 3)	6(8. 0)	0. 175
Recurrent myocardial infarction^,c^	21(7. 0)	2(2. 7)	4(5. 3)	6(8. 0)	9(12. 0)	0. 012
Acute heart failure^,c^	29(9. 7)	2(2. 7)	5(6. 7)	8(10. 7)	14(18. 7)	0. 002
Cardiogenic shock^,c^	14(4. 7)	2(2. 7)	2(2. 7)	4(5. 3)	6(8. 0)	0. 157

MACE, Major Adverse Cardiovascular Events, defined as the composite endpoint of cardiac death, acute stent thrombosis, recurrent myocardial infarction, acute heart failure, and cardiogenic shock. Data are presented as number of cases (percentage). ^c^Categorical variables were expressed as frequency (percentage) and compared using chi-square test or Fisher’s exact test.

**Table 3** shows the results of multivariate logistic regression analysis of the relationship between CTI and in-hospital MACE. In the unadjusted model (Model 1), compared with the Q1 group, the risk of MACE gradually increased in the Q2, Q3, and Q4 groups (Q2, OR 1. 54, 95%CI 0. 61-3. 89, P , 0. 364; Q3, OR 2. 74, 95%CI 1. 17-6. 42, P , 0. 020; Q4, OR 5. 06, 95%CI 2. 25-11. 39, P<0. 001). After adjusting for demographic factors (Model 2), this association was slightly attenuated but remained significant. After full adjustment for clinical, laboratory, and coronary angiography characteristics (Model 3), the highest CTI quartile group (Q4) remained significantly associated with MACE risk (OR 3. 28, 95%CI 1. 42-7. 56, P , 0. 005). Additionally, when analyzing CTI as a continuous variable, each standard deviation increase in CTI was associated with a 46% increase in MACE risk (OR 1. 46, 95%CI 1. 21-1. 76, P<0. 001).

**Table 3:** Multivariate logistic regression analysis of the relationship between CTI and MACE during hospitalization.

CTI Group	Model 1		Model 2		Model 3	
	OR (95% CI)	P-value	OR (95% CI)	P-value	OR (95% CI)	P-value
Q1	1. 00(Reference)	–	1. 00(Reference)	–	1. 00(Reference)	–
Q2	1. 54 (0. 61-3. 89)	0. 364	1. 47 (0. 58-3. 74)	0. 420	1. 31 (0. 51-3. 38)	0. 578
Q3	2. 74 (1. 17-6. 42)	0. 020	2. 51 (1. 05-5. 97)	0. 038	2. 16 (0. 89-5. 25)	0. 089
Q4	5. 06 (2. 25-11. 39)	<0. 001	4. 38 (1. 91-10. 06)	<0. 001	3. 28 (1. 42-7. 56)	0. 005
Continuous CTI (per SD increase)	1. 68 (1. 42-1. 98)	<0. 001	1. 59 (1. 34-1. 89)	<0. 001	1. 46 (1. 21-1. 76)	<0. 001

OR, Odds Ratio; CI, Confidence Interval; SD, Standard Deviation.

Model 1, Unadjusted.

Model 2, Adjusted for age, sex, and BMI.

Model 3, Adjusted for age, sex, BMI, hypertension, diabetes, smoking, history of MI, ACS type, LVEF, multivessel disease, complex lesions (Type B2/C), number of stents, LDL-C, HDL-C, and eGFR.

### 3.3 Non-linear relationship between CTI and in-hospital MACE

**Figure 2:**
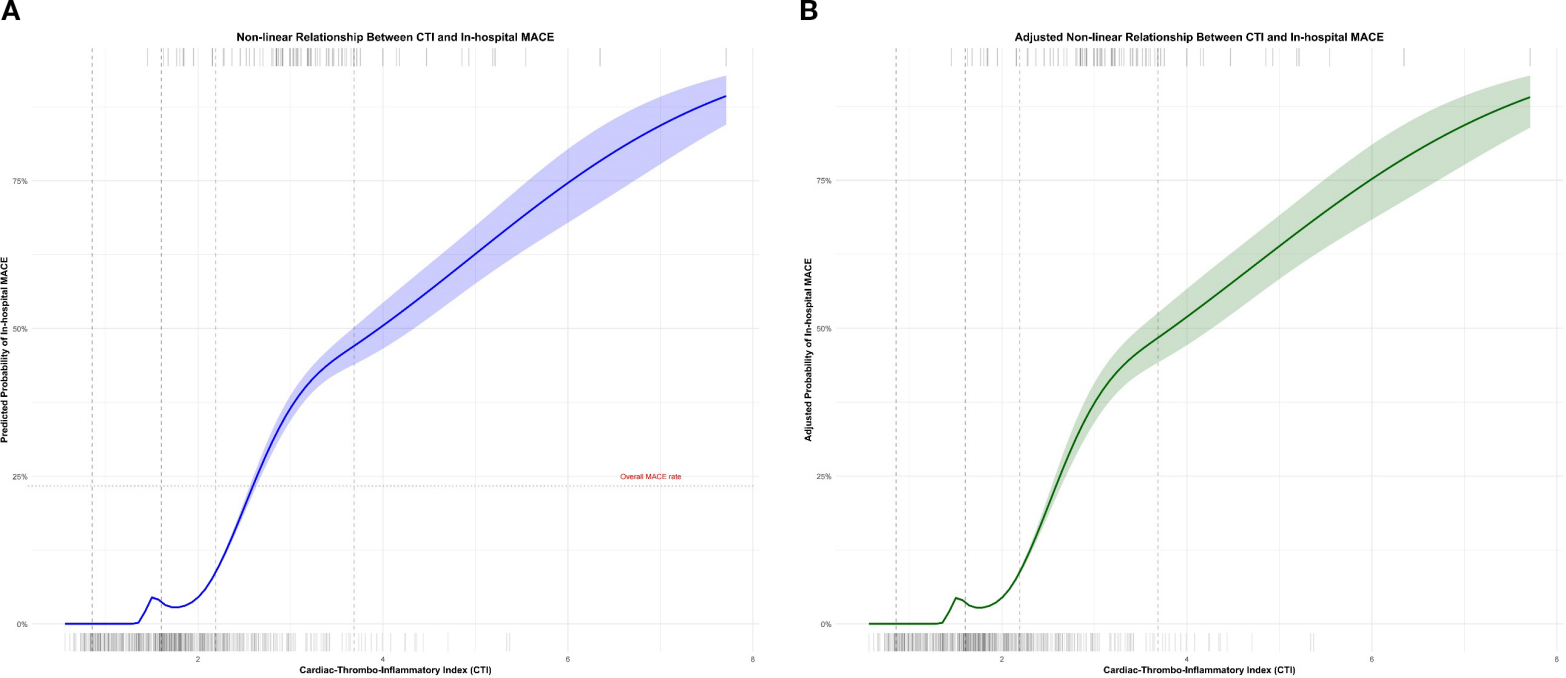
Non-linear relationship between C-Reactive Protein-Triglyceride-Glucose Index(CTI) and in-hospital Major Adverse Cardiovascular Events (MACE). **(A)** The blue solid line represents the estimated probability of in-hospital MACE derived from restricted cubic spline analysis, with the light blue shaded area representing the 95% confidence interval. Vertical dashed lines mark the knot positions (placed at the 5th, 35th, 65th, and 95th percentiles of the CTI distribution). The short lines at the top of the graph indicate the distribution of CTI values in patients who experienced MACE, while those at the bottom represent patients without MACE. The red horizontal dotted line indicates the overall MACE incidence rate in the entire cohort (24. 3%). **(B)** This non-linear relationship remained significant after adjusting for covariates (p, 0. 023).

Restricted cubic spline analysis revealed a significant non-linear relationship between CTI values and the probability of in-hospital MACE (p for non-linearity , 0. 018) (**Figure 2**). The risk of MACE remained relatively low and increased gradually at CTI values below 1. 8. Between CTI values of approximately 1. 8 and 3. 2, there was a steeper increase in MACE risk, indicating an accelerated risk accumulation within this range. At very high CTI values (>3. 2), the slope became less steep, suggesting a potential plateau effect, though the confidence intervals also widened at these extreme values due to fewer observations. This non-linear pattern remained significant after adjusting for age, sex, BMI, hypertension, diabetes, smoking, history of MI, ACS type, LVEF, multivessel disease, complex lesions (Type B2/C), number of stents, LDL-C, HDL-C, and eGFR(p for non-linearity , 0. 023).

### 3.4 Predictive value of CTI

**Figure 3:**
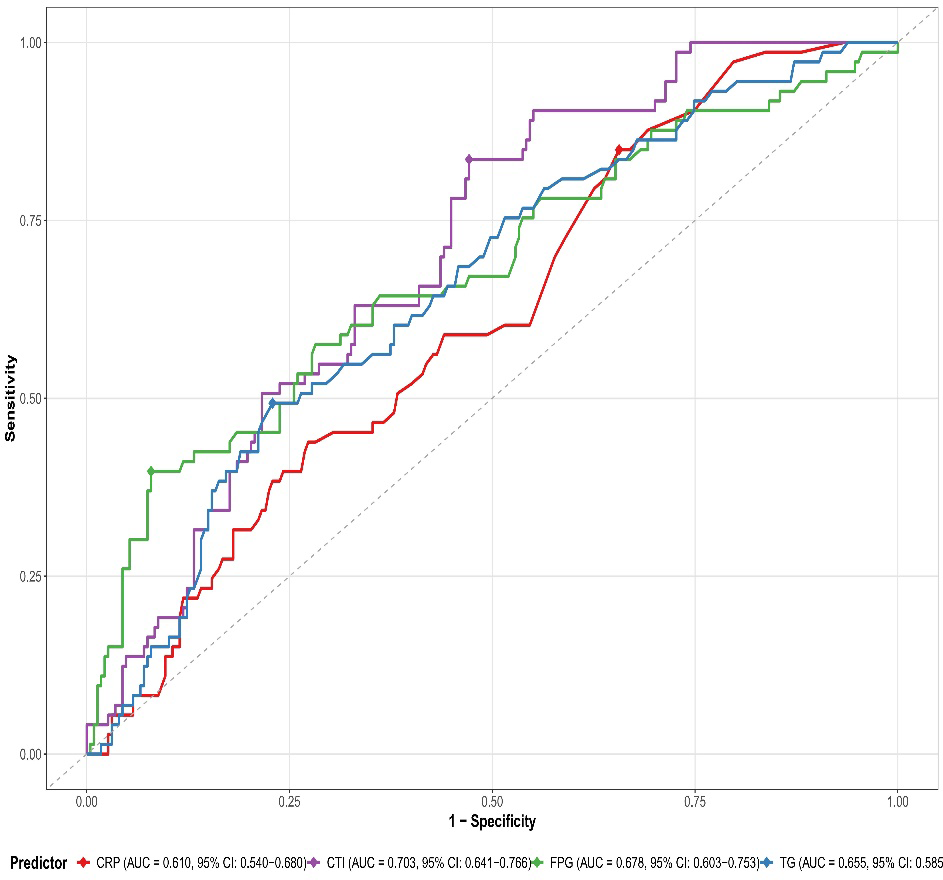
Receiver Operating Characteristic (ROC) curve analysis comparing the predictive performance of CTI and conventional biomarkers for in-hospital Major Adverse Cardiovascular Events (MACE) in patients with Acute Coronary Syndrome. The graph displays ROC curves for CTI (C-Reactive Protein-Triglyceride-Glucose Index; solid purple line), CRP (C-Reactive Protein; dashed red line), FPG (Fasting Plasma Glucose; dotted green line), and TG (Triglycerides; dash-dotted blue line).

ROC curve analysis showed (**Figure 3**) that the AUC of CTI for predicting in-hospital MACE was 0. 703 (95%CI, 0. 641-0. 766), significantly higher than that of CRP alone (AUC, 0. 610, 95%CI, 0. 540-0. 680, P<0. 001), TG alone (AUC, 0. 655, 95%CI, 0. 585-0. 725, P<0. 001), and FPG alone (AUC, 0. 678, 95%CI, 0. 603-0. 753, P , 0. 003).

The optimal cut-off values and corresponding diagnostic parameters for predicting in-hospital MACE are presented in **Table 4**. For CTI, a cut-off value of 3. 42 provided sensitivity of 71. 8%, specificity of 68. 9%, positive predictive value of 45. 7%, negative predictive value of 87. 1%, and accuracy of 69. 7%. For CRP, a cut-off value of 8. 74 mg/L yielded sensitivity of 63. 4%, specificity of 58. 6%, positive predictive value of 34. 8%, negative predictive value of 82. 2%, and accuracy of 60. 0%. The optimal cut-off value for TG was 1. 82 mmol/L, with sensitivity of 68. 3%, specificity of 62. 4%, positive predictive value of 38. 2%, negative predictive value of 85. 1%, and accuracy of 64. 0%. For FPG, a cut-off value of 6. 93 mmol/L demonstrated sensitivity of 69. 1%, specificity of 66. 5%, positive predictive value of 41. 5%, negative predictive value of 86. 3%, and accuracy of 67. 3%.

**Table 4:** Diagnostic parameters of CRP, TG, FPG, and CTI for predicting in-hospital MACE.

Variables	Cut-off	Sensitivity	Specificity	PPV	NPV	Accuracy
CTI	3. 42	71. 8%	68. 9%	45. 7%	87. 1%	69. 7%
CRP(mg/L)	8. 74	63. 4%	58. 6%	34. 8%	82. 2%	60. 0%
TG(mmol/L)	1. 82	68. 3%	62. 4%	38. 2%	85. 1%	64. 0%
FPG(mmol/L)	6. 93	69. 1%	66. 5%	41. 5%	86. 3%	67. 3%

MACE, Major adverse cardiovascular events; CTI, C-reactive protein-triglyceride-glucose index; CRP, C-Reactive Protein; TG, Triglyceride; FPG, Fasting plasma glucose; PPV, Positive predictive value; NPV, Negative predictive value.

**Table 5** shows the incremental predictive value of CTI over traditional risk models. Compared with the baseline model containing only traditional risk factors, the model with added CTI showed significantly improved AUC (0. 745 *vs* 0. 682, P , 0. 015). Furthermore, after adding CTI, the NRI and IDI were 0. 213 (95%CI 0. 107-0. 319, P<0. 001) and 0. 036 (95%CI 0. 016-0. 056, P<0. 001) respectively, indicating that CTI significantly improved risk prediction capability.

**Table 5:** Assessment of the incremental value of CTI for predicting MACE during hospitalization.

Models	AUC (95% CI)	P-value	NRI (95% CI)	P-value	IDI (95% CI)	P-value
CTI	0. 703(0. 641-0. 766)	–	–	–	–	–
Grace Score	0. 636(0. 588-0. 684)	Reference	Reference	–	Reference	–
Baseline Model*	0. 682(0. 630-0. 734)	Reference	Reference	–	Reference	–
Grace Score+CTI	0. 713(0. 651-0. 788)	0. 009	0. 156(0. 084-0. 228)	<0. 001	0. 025(0. 012-0. 038)	<0. 001
Baseline Model+CTI	0. 745(0. 697-0. 793)	0. 015	0. 213(0. 107-0. 319)	<0. 001	0. 036(0. 016-0. 056)	<0. 001

AUC, Area Under the Receiver Operating Characteristic Curve; NRI, Net Reclassification Improvement; IDI, Integrated Discrimination Improvement; CI, Confidence Interval. *The baseline model includes age, sex, BMI, hypertension, diabetes, smoking, history of MI, ACS type, LVEF, multivessel disease, complex lesions, number of stents, LDL-C, HDL-C, and eGFR.

### 3.5 Subgroup analysis

**Table 6** shows the adjusted ORs for prediction of in-hospital MACE by CTI in different subgroups (comparing Q4 with Q1 group). In all subgroups, high CTI was associated with increased risk of MACE, but this association was stronger in certain subgroups. In particular, the association between CTI and MACE was more significant in patients with STEMI (OR 4. 62, 95%CI 1. 52-14. 06), diabetes (OR 4. 83, 95%CI 1. 59-14. 65), and multivessel disease (OR 3. 96, 95%CI 1. 46-10. 76). Interaction analysis indicated that ACS type (P interaction, 0. 039) and diabetes status (P interaction, 0. 046) had significant moderating effects on the relationship between CTI and MACE.

**Table 6:** Subgroup analysis of the impact of high CTI (Q4 Group) versus low CTI (Q1 Group) on MACE risk in different subgroups.

Subgroup	Sample size(n)	Number of events/total	Adjusted OR(95% CI)	P-value	Interaction p-value
Total	300	73/300	3. 28 (1. 42-7. 56)	0. 005	–
Age					0. 262
<65,years	158	32/158	2. 83 (0. 98-8. 21)	0. 055	
≥65,years	142	41/142	3. 65 (1. 28-10. 42)	0. 015	
Gender					0. 384
Male	202	46/202	2. 94 (1. 15-7. 53)	0. 025	
Female	98	27/98	3. 79 (1. 08-13. 29)	0. 037	
BMI					0. 715
<25,kg/m²	149	31/149	3. 12 (1. 05-9. 28)	0. 041	
≥25,kg/m²	151	42/151	3. 41 (1. 21-9. 61)	0. 020	
Diabetes					0. 046
Yes	97	33/97	4. 83 (1. 59-14. 65)	0. 005	
No	203	40/203	2. 35 (0. 85-6. 47)	0. 099	
ACS Types					0. 039
STEMI	103	36/103	4. 62 (1. 52-14. 06)	0. 007	
NSTEMI/UA	197	37/197	2. 17 (0. 78-6. 04)	0. 138	
Vessel Disease					0. 082
Single Vessel	143	25/143	2. 48 (0. 78-7. 89)	0. 124	
Multiple Vessels	157	48/157	3. 96 (1. 46-10. 76)	0. 007	

OR, Odds Ratio; CI, Confidence Interval; BMI, Body Mass Index; ACS, Acute Coronary Syndrome; STEMI, ST-Elevation Myocardial Infarction; NSTEMI, Non-ST-Elevation Myocardial Infarction; UA, Unstable Angina. *Adjusted OR represents the MACE risk of the fourth quartile CTI group (Q4, ≥1. 34) compared to the first quartile group (Q1, <0. 62), adjusted for age, sex, BMI, hypertension, diabetes, smoking, history of MI, ACS type, LVEF, multivessel disease, complex lesions, number of stents, LDL-C, HDL-C, and eGFR (excluding the respective variable when analyzing the corresponding subgroup).

### 3.6 Comparison of CTI with GRACE score

ROC curve analysis revealed that CTI (AUC, 0. 703, 95% CI, 0. 641-0. 766) demonstrated comparable discriminatory power to the GRACE score (AUC, 0. 636, 95% CI, 0. 588-0. 684, P , 0. 682 for comparison) for predicting in-hospital MACE. However, when CTI was added to the GRACE score, there was a significant improvement in risk prediction (AUC for combined model, 0. 713, 95% CI, 0. 651-0. 788, P , 0. 009 versus GRACE score alone). The addition of CTI to the GRACE score resulted in significant net reclassification improvement (NRI , 0. 356, 95% CI, 0. 184-0. 528, P < 0. 001) and integrated discrimination improvement (IDI , 0. 085, 95% CI, 0. 042-0. 128, P < 0. 001),indicating that CTI provided incremental prognostic value beyond the GRACE score (**Table 5**).

## 4 Discussion

This study investigated the association between the C-reactive protein-triglyceride-glucose (CTI) index and in-hospital major adverse cardiovascular events (MACE) in acute coronary syndrome patients after PCI treatment. The results showed that elevated CTI levels were significantly associated with increased risk of in-hospital MACE in ACS patients after PCI, and this association persisted after adjusting for traditional risk factors. This finding emphasizes the potential value of a composite index integrating inflammatory and metabolic markers in predicting short-term prognosis in ACS patients.

Our study demonstrated that with increasing CTI quartiles, the incidence of MACE showed a gradient upward trend, rising from 10. 7% in the lowest quartile group to 44. 0% in the highest quartile group. This association remained significant in multivariate analysis, suggesting that CTI may be an independent predictor of adverse prognosis during hospitalization for ACS patients. This is consistent with previous studies on the relationship between individual inflammatory or metabolic markers and cardiovascular events (37, 38). Ridker et al. demonstrated that elevated CRP levels are an independent predictor of recurrent cardiovascular events in patients with acute coronary events (39). Similarly, Miller et al. found in the PROVE IT-TIMI 22 study that high TG levels were associated with increased risk of recurrent cardiovascular events in ACS patients (12). A meta-analysis by Capes et al. confirmed that stress hyperglycemia was significantly associated with increased in-hospital mortality in patients with acute myocardial infarction (13). Our study further extends these findings, suggesting that the CTI index, which integrates these markers, may provide a more comprehensive risk assessment than individual indicators.

CTI, as a novel composite index, integrates information from three aspects, CRP reflecting inflammatory status, TG reflecting lipid metabolism, and FPG reflecting glucose metabolism. ROC curve analysis in this study showed that the AUC of CTI for predicting in-hospital MACE was 0. 703, significantly higher than that of CRP, TG, and FPG alone, indicating the superiority of composite indicators in risk prediction. This result is consistent with studies on other composite indicators. For example, Park et al. found that the CRP/albumin ratio, which integrates CRP and albumin, was superior to CRP alone in predicting in-hospital death in patients with acute myocardial infarction (40). Similarly, Li et al. demonstrated that the triglyceride-glucose index showed good predictive value in assessing coronary heart disease severity (41). Chen et al. showed a significant association between C-reactive protein-triglyceride glucose index and new onset coronary heart disease in metabolically heterogeneous individuals (42). These studies collectively support that composite biomarkers may provide more comprehensive and accurate risk assessment.

Our study demonstrates that CTI offers several advantages compared to traditional risk scores such as the GRACE score. First, while the GRACE score primarily focuses on clinical parameters and has limited incorporation of inflammatory markers, CTI directly integrates multiple pathophysiological pathways involved in ACS, including inflammation, thrombosis, and metabolic dysregulation. This comprehensive approach may better reflect the complex pathophysiology underlying adverse events in ACS patients. Second, our results show that CTI provides incremental prognostic value when added to the GRACE score, as evidenced by significant improvements in NRI and IDI. This suggests that CTI captures additional risk information not contained within the GRACE score, potentially enabling more accurate risk stratification. Third, CTI is composed of laboratory markers that are routinely available in most hospital settings, making it practical for widespread clinical implementation without requiring additional specialized testing. While the GRACE score includes variables that may not be immediately available upon presentation (e. g., serum creatinine for calculation of eGFR), the components of CTI are typically included in the standard admission laboratory panel for ACS patients. However, we acknowledge that CTI requires external validation in larger, diverse populations before it can be recommended for routine clinical use. Additionally, the calculation of CTI involves multiple biomarkers, which might be seen as more complex than single-marker approaches. Nevertheless, the potential improvements in risk stratification may justify this additional complexity.

Notably, our study also confirmed that CTI provides significant incremental predictive value to traditional risk models. After adding CTI, the AUC of the risk prediction model increased significantly from 0. 682 to 0. 745, with significant improvements in both NRI and IDI, indicating that CTI can provide additional prognostic information to existing risk assessment tools. This is consistent with the study by Mjelva et al., which found that adding inflammatory markers to traditional risk prediction models can significantly improve the accuracy of risk stratification in ACS patients (43). Similarly, Li et al. also showed that adding biomarkers to the GRACE score can improve the predictive ability for in-hospital events in ACS patients (44). These results support the use of CTI as a complement to traditional risk assessment in clinical practice.

Subgroup analysis results showed that the association between CTI and MACE was more significant in STEMI patients, diabetic patients, and patients with multivessel disease. This difference may reflect the special pathophysiological significance of inflammatory responses and metabolic disorders in these subgroups. STEMI patients typically present with larger areas of myocardial damage and more severe inflammatory responses (45). Diabetic patients have more complex metabolic abnormalities, including insulin resistance, hyperglycemia, and lipid metabolism disorders, which collectively promote endothelial dysfunction, inflammatory activation, and atherosclerosis progression (46). Klingenberg et al. found that diabetic patients with ACS have higher levels of inflammatory markers, which are associated with worse prognosis (47). Multivessel disease reflects more extensive coronary atherosclerosis and greater atherosclerotic burden; these patients often have more severe systemic inflammation and metabolic disorders (48). Palmerini et al. confirmed that patients with multivessel disease have increased risk of complications after PCI and poorer prognosis (49). Our subgroup analysis results emphasize the potential clinical value of integrating inflammatory and metabolic markers in these high-risk populations.

The potential mechanisms of the association between CTI and MACE may involve the synergistic effects of inflammatory responses and metabolic disorders in coronary atherosclerotic plaque instability and post-PCI myocardial injury. CRP is not only a marker of inflammation but can also directly participate in atherosclerosis progression through multiple mechanisms, including promoting monocyte adhesion and chemotaxis, activating the complement system, enhancing oxidative stress, and inducing endothelial dysfunction (23, 50). High TG levels can increase the formation of small dense LDL particles, promote endothelial dysfunction and inflammatory responses, and increase the risk of thrombosis (24). Hyperglycemia damages vascular endothelial cells and myocardial cells through mechanisms such as advanced glycation end-product formation, increased oxidative stress, and activation of inflammatory cascade reactions (25). Ceriello et al. proposed the concept of “metabolic memory,” suggesting that short-term hyperglycemia can lead to persistent vascular damage and inflammatory responses through epigenetic modifications (51). These factors working together may explain why patients with higher CTI have a higher risk of MACE.

Additionally, we observed that patients in higher CTI groups had more multivessel disease and complex lesion characteristics, suggesting that CTI may reflect the extent and complexity of coronary atherosclerosis. Gensini et al. demonstrated that the severity of coronary artery lesions positively correlates with levels of inflammatory markers such as CRP (52). These findings support CTI as a potential marker reflecting the severity and complexity of coronary artery disease. It is worth discussing that in this study, CTI calculation was based on CRP, TG, and FPG levels measured at admission, which may be influenced by acute phase reactions and medication. For example, some patients may have already received statin therapy, affecting TG levels; similarly, acute stress responses can affect FPG levels (53). However, admission test results have strong clinical utility as they are the earliest available information in the decision-making process. Moreover, previous studies have shown that the predictive value of these markers persists even in the acute phase (8). Future research could consider evaluating the predictive value of CTI measured at different time points to determine the optimal assessment timing. In recent years, multiple studies have explored the application of comprehensive scoring systems integrating multiple biomarkers in cardiovascular disease prediction. Our study provides new evidence for this field, supporting that integrating inflammatory and metabolic markers may be an effective strategy to improve risk prediction accuracy.

From a clinical application perspective, CTI has several significant advantages, First, the three indicators constituting CTI (CRP, TG, and FPG) are all routine examination items, requiring no additional testing, making them cost-effective and easily accessible; Second, CTI calculation is simple and can be conveniently applied in clinical practice; Third, CTI provides more comprehensive risk assessment information than single indicators. Therefore, CTI can serve as a simple and practical tool for identifying high-risk ACS patients after PCI, guiding more aggressive treatment strategies and close monitoring. For example, patients with higher CTI may require more intensive statin therapy, stricter glycemic control, and closer follow-up monitoring (54). This is consistent with the concept of “residual inflammatory risk” proposed by Libby et al., emphasizing treatment strategies targeting inflammation and metabolic disorders on the basis of controlling traditional risk factors (55).

Despite our study providing valuable findings, several limitations exist. First, as a single-center prospective observational study with a relatively modest sample size (n, 300), our findings may have limited generalizability to broader populations. Although our study achieved adequate statistical power (>90%) to detect significant differences in MACE rates across CTI quartiles, and each quartile group (n, 75) was sufficient for the primary comparative analysis, the observational design cannot establish a causal relationship between CTI and MACE. Larger multicenter studies are warranted to validate our findings and enhance external validity. Second, we only evaluated short-term prognosis during hospitalization and have not clarified the predictive value of CTI for long-term outcomes. Third, we were unable to assess the relationship between dynamic changes in CTI and prognosis, and a single measurement may not fully reflect the patient’s metabolic and inflammatory status. Fourth, the sample size was relatively limited in some subgroup analyses.

In conclusion, this study demonstrates that CTI is an independent predictor of in-hospital MACE in ACS patients after PCI, with predictive capability superior to CRP, TG, or FPG alone. CTI provides significant incremental predictive value over traditional risk models, particularly in patients with STEMI, diabetes, and multivessel disease. As a simple indicator integrating inflammatory and metabolic information, CTI has the potential to become a useful tool for risk stratification in clinical practice, guiding individualized treatment strategies. Future large-scale prospective studies are needed to further validate the predictive value of CTI and explore whether CTI-based intervention strategies can improve outcomes in ACS patients.

## Data Availability

The original contributions presented in the study are included in the article/supplementary material. Further inquiries can be directed to the corresponding authors.
